# Phenazopyridine-Induced Methemoglobinemia: A Case Report

**DOI:** 10.7759/cureus.33715

**Published:** 2023-01-12

**Authors:** Alexis A Koch, Laurence Stolzenberg, Prutha R Pathak, Alexis R Penot

**Affiliations:** 1 Internal Medicine, Alabama College of Osteopathic Medicine, Dothan, USA; 2 Internal Medicine, Decatur Morgan Hospital, Decatur, USA

**Keywords:** case report, methylene blue, arterial blood gas, urinary tract infection, phenazopyridine, toxicology, acquired methemoglobinemia

## Abstract

Methemoglobinemia is a condition caused by increased methemoglobin, a reduced form of hemoglobin, in the blood. This causes the molecules to bind oxygen more tightly and decreases their ability to release that oxygen to tissue. Most cases of methemoglobinemia are acquired and occur either in pediatric populations or in individuals with predisposing conditions. This report illustrates a case of an otherwise healthy 31-year-old patient presenting to the emergency department with cyanosis of the hands and mouth and an O2 saturation of 78% after taking increased doses of the over-the-counter medication phenazopyridine. A “chocolate-brown” color of her arterial blood, and increased methemoglobin levels of 20.2%, confirmed the diagnosis of methemoglobinemia. She was treated with both methylene blue and ascorbic acid, and her oxygen saturation and serum chemistry returned to normal levels within a few hours. The case highlights the importance of discussing the dosage of all over-the-counter medications with patients and recognizing the signs and symptoms of methemoglobinemia.

## Introduction

Oxygen transport in the body relies on the proper function of hemoglobin proteins. Each individual hemoglobin contains an iron molecule at its center in a ferrous (Fe^2+^) state. This ferrous state allows the hemoglobin to both bind oxygen in the lungs and release it to the tissues for normal physiologic function. However, when that iron is oxidized to its ferric state (Fe^3+^), it binds oxygen more tightly, and its ability to release that oxygen to tissues is decreased. This oxidized form of hemoglobin is called methemoglobin. If there are too many methemoglobin molecules in the blood, a condition known as methemoglobinemia causes hypoxemia in tissues and can become fatal if left untreated. Levels of 15% methemoglobin in the blood cause cyanosis of skin and oral mucosa. If levels increase to higher than 15%, it can cause symptoms of hypoxia and various neurological and cardiac symptoms such as headache, altered mental status, seizures, coma, dyspnea, heart palpitations, chest pain, cardiac arrhythmias, and myocardial infarction. Levels above 70% are generally fatal [1,2].

Methemoglobinemia can be either congenital or acquired. Acquired methemoglobinemia is more common in infants and is most frequently caused by exposure to local anesthetics, dapsone, and nitrates or nitrites [3]. Less typically, but still importantly, methemoglobinemia can be caused by other common sources, including, e-cigarettes, cocaine or other 'street' drugs cut with benzocaine, inhalations of butyl nitrate ‘poppers,’ and ingestions of shoe dye or polish [4-7]. These substances act as oxidants and cause the reduction of hemoglobin to its methemoglobin form. Genetic risk factors such as a deficiency in cytochrome b5 reductase or glucose 6-phosphate dehydrogenase (G6PD) can cause increased susceptibility to developing this condition. Dapsone and Benzocaine are among the most cited causes in the general population [8]. Phenazopyridine is a rare cause of methemoglobinemia, especially in adults without any predisposing conditions [9]. Cases of acquired methemoglobinemia in adults are considered a medical emergency.

The treatment of methemoglobinemia consists of first stopping the precipitating agent and providing supportive therapy, including supplemental oxygen and intravenous (IV) hydration. Methylene blue, originally introduced as a textile dye in 1876, is now the primary treatment for reducing the ferric iron of methemoglobin back to its ferrous state [10]. It is initially administered intravenously at a rate of 1-2 mg/kg (0.2 mL/kg of a 1% solution) for 3 to 5 minutes for an adult dose [2,11]. Its effects usually occur 30-60 minutes after administration. The most common side effects of methylene blue include abdominal pain, nausea and blue discoloration of the skin and/or urine [12]. However, it can cause severe hemolytic complications in individuals with G6PD deficiency [12]. Additionally, methylene blue should be avoided in patients taking a monoamine oxidase inhibitor as it may elicit serotonin syndrome [12]. In these cases, hyperbaric oxygen, ascorbic acid, or Vitamin C, longer-acting reducing agents, can be used as alternative treatments [13-15].

## Case presentation

A 31-year-old female presented to the Emergency Department with weakness and cyanosis of her hands and lips. She noticed her lips were blue that morning but did not pay much attention to it and continued with her daily activities until she was alerted by her smartwatch that her O_2_ saturation was low and eventually decided to go to the emergency department that evening. During history, she reported feeling fine other than the blue lips and fingers and otherwise denied any chest pain, shortness of breath, leg swelling, cough, fevers, chills, or significant abdominal pain. She did admit to having dysuria the past few days and had been taking several doses of phenazopyridine to treat her symptoms. She had no significant medical history and denied any history of blood clots, recent travel, recent surgery, and any carbon monoxide exposure. She was taking no prescribed or other over-the-counter medications. She drank alcohol occasionally but denied any current or past tobacco or illicit drug use.

A physical exam showed a female in no respiratory distress but revealed cyanosis of her lips and her hands. Her presenting O_2_ saturation was 78% on room air, which only improved to 86% following the administration of a 15L nonrebreather mask. Their vitals were otherwise unremarkable, and the rest of her physical exam was nonrevealing. She had no tachypnea, accessory muscle use, wheezing, or coughing. Auscultation revealed clear breath sounds. The auscultation of her heart sounds revealed no murmurs, gallops, or rubs. Her abdomen was soft and non-tender, and there was no lower extremity edema.

At this point, a differential between acute coronary syndrome (ACS), pulmonary embolus (PE), pneumonia, and drug overdose was explored. Laboratory investigations revealed a white cell count of 13,500 white blood cells (WBCs) per microliter. Hemoglobin and hematocrit were normal at 14.6 g/dL and 45.8 g/dL. Phosphorus was low at 1.6mg/dL. Urinalysis was positive for nitrates, bilirubin, WBCs, and leukocytes. Toxicology was positive for cannabinoids. CT pulmonary angiogram showed no evidence of pulmonary emboli. Chest X-ray and echocardiogram were normal. Arterial blood gas consisted of "chocolate-colored" blood from the arterial side and revealed a pH of 7.57, pCO_2_ of 23 mmHg, PO_2_ of 188 mmHg on 100% FiO_2_, with methemoglobin of 20.2%, and a lactate of 2.4 mg/dL.

Based on the arterial blood gas (ABG) results, the diagnosis of methemoglobinemia was then made. Pulmonology and toxicology were consulted, and a dose of methylene blue was administered. Due to concern for possible G6PD deficiency, a blood sample was sent out for analysis. The patient and her mother denied any family history of G6PD deficiency; however, precautions and adverse outcomes from the methylene blue were considered before administration. While some patients with methemoglobinemia can be watched and may improve on their own, it was decided that given her persistent hypoxia and cyanosis, treatment should be administered. A dose of 1mg/kg of methylene blue was co-administered with 500mg ascorbic acid in case the patient was G6PD deficient.

Following administration of the methylene blue, the patient experienced nausea and had multiple episodes of emesis followed by improvement in her O_2_ saturation and cyanosis. A repeat ABG was done four hours later and showed complete resolution of the methemoglobinemia with a concentration of 0.7%. Acid-base abnormalities were still present but resolved with supportive intervention over the next few hours. The next morning, methemoglobin levels had dropped further to 0.4, her blood chemistry normalized, and her oxygen saturation remained around 96% on room air.

Urine culture revealed a urinary tract infection of *E. coli*, and the patient was discharged the next day on appropriate antibiotics and has remained symptom-free. G6PD results came back within normal range, and the patient was counseled on avoiding phenazopyridine in the future.

## Discussion

Phenazopyridine-induced methemoglobinemia is a rare condition, especially in healthy adults with no history of enzyme deficiencies. Specific compounds are known to induce methemoglobinemia, but due to individual differences in metabolic pathways, it is difficult to predict when and at what dosage they might cause the complication. Past medical or family history of cytochrome b5 reductase or glucose 6-phosphate dehydrogenase (G6PD) deficiency should raise automatic suspicion for methemoglobinemia upon presentation with cyanosis; however, absence of these genetic risk factors should not remove this condition from a differential diagnosis. It is important to consider methemoglobinemia on presentation of cyanosis and hypoxia unresponsive to supplemental oxygen and other signs and symptoms that may initially cause concern for pulmonary embolisms or cardiac syndromes in otherwise healthy individuals.

Ascorbic acid is an effective alternative to methylene blue in the treatment of methemoglobinemia. It works as an oxidizer but acts slowly. The recommendations for dosage and duration for treatment with ascorbic acid use are inconsistent in literature and vary widely [16]. In patients with G6PD deficiency, methylene blue is contraindicated as it can cause a hemolytic crisis, and ascorbic acid is then indicated as the sole treatment. If available, hyperbaric oxygen chambers are recommended as they may improve the effectiveness of treatment and accelerate the reduction of methemoglobin [14,15]. The efficacy of using both methylene blue and ascorbic acid as a first-line treatment has not been well studied, but there are few side effects and contraindications for the supplementation of ascorbic acid in patients that can tolerate methylene blue [16]. Especially if methylene blue is not available for treatment, ascorbic acid should be considered [17].

Methemoglobinemia due is a rare condition, with only 100 cases of total methemoglobinemia over the past five years reported to the American Association of Poison Control. However, the incidence is likely underestimated due to physicians or providers not notifying appropriate poison control centers when cases arise [13,18,19].

Phenazopyridine is a known inducer of methemoglobinemia, and it is important to ask about the use and quantity of over-the-counter medications used, especially for the treatment of urinary tract symptoms, and then contacting the appropriate poison control centers and personnel when the diagnosis is made.

## Conclusions

Acquired methemoglobinemia is an uncommon condition in adults with no predisposing conditions and can be clinically difficult to recognize and diagnose. Additionally, it is a rare side effect of phenazopyridine. Patients may unwittingly over-use this uncontrolled medication, resulting in methemoglobinemia. It is, therefore, critical for healthcare practitioners to conduct a detailed history and always ask for all over-the-counter medications used by a patient and the quantity taken. It is also important to keep this diagnosis in the differential for patients presenting with hypoxemia and cyanosis so they can be treated swiftly and effectively.
